# The effect of intratympanic gentamicin as a prehabilitation strategy for objective and subjective vestibular function in patients undergoing microsurgery for a unilateral vestibular schwannoma

**DOI:** 10.1007/s00405-023-08240-1

**Published:** 2023-09-26

**Authors:** Constanza Fuentealba Bassaletti, Babette F. van Esch, Jeroen C. Jansen, Peter Paul G. van Benthem, Erik F. Hensen

**Affiliations:** 1 Department of Otorhinolaryngology and Head and Neck Surgery, Leiden University Medical Centre, Albinusdreef 2, 2333 ZA Leiden, The Netherlands; 2 Escuela de Fonoaudiología, Facultad de Odontología y Ciencias de la Rehabilitación, Universidad San Sebastián, Santiago, Chile

**Keywords:** Vestibular schwannoma, Intratympanic gentamicin, Prehabilitation, Vestibular function, Objective vestibular test, Vestibular symptoms

## Abstract

**Objective:**

To review the literature on intratympanic gentamicin treatment as prehabilitation for patients undergoing surgery for a unilateral vestibular schwannoma.

**Data sources:**

A systematic literature search was conducted up to March 2023 in Pubmed, Embase, Cochrane, Web of Science, Academic Search Premier, Google Scholar and Emcare databases.

**Review methods:**

Articles on the effect of intratympanic gentamicin followed by vestibular schwannoma surgery were reviewed. Data on objective vestibular function and subjective outcomes were compiled in tables for analysis. Relevance and methodological quality were assessed with the methodological index for non-randomized tool.

**Results:**

A total of 281 articles were identified. After screening and exclusion of duplicates, 13 studies were reviewed for eligibility, of which 4 studies could be included in the review. The posturography test, the subjective visual horizontal test, and the optokinetic nystagmus test showed decreased vestibular function in the group of patients who received intratympanic gentamicin before microsurgery compared to the group of patients without gentamicin. Other objective tests did not show significant differences between patient groups. Subjective vestibular outcomes, as evaluated by questionnaires on quality of life and/or dizziness, did not seem to improve from intratympanic gentamicin pretreatment.

**Conclusion:**

Vestibular schwannoma patients who received intratympanic gentamicin before surgical resection of the tumor performed better in the posturography test, subjective visual horizontal test, and the optokinetic nystagmus test afterwards. However, studies that also evaluated subjective outcomes such as dizziness, anxiety, depression, and balance self-confidence did not show a positive effect of intratympanic gentamicin on the vestibular complaints and symptoms.

## Introduction

A significant number of patients with unilateral vestibular schwannoma (VS) suffer from imbalance, dizziness, or vertigo during the natural course of the disease or after treatment [1]. In some cases, the vestibular symptoms may decrease or resolve over time. It is hypothesized that this is the effect of central compensation. However, it has been reported that a proportion of VS patients suffer from vestibular symptoms in the long term, impacting on their long-term quality of life [2]. This may be because the vestibular compensation is insufficient, or because of other factors that affect dizziness and balance in this patient group. The preoperative chemical ablation with intratympanic gentamicin (ITG) aims to gradually deteriorate the ipsilateral labyrinth function before surgical removal of the vestibular schwannoma, to ameliorate the vestibular symptoms or accelerate vestibular rehabilitation after surgery [3]. Schuknecht was the first to publish on the potential effect of aminoglycosides (streptomycin) resulting in vestibular chemical ablation in patients with Menière’s disease. To preserve the patient’s hearing, the use of gentamicin is preferred due to its vestibulotoxicity properties [4, 5]. Magnusson et al. coined the concept of vestibular prehabilitation, i.e., vestibular rehabilitation exercises combined with ITG treatment before surgery. It was aimed to trigger the vestibular re-programming in advance to the surgical ablation [6]. This process leaves the patient with a gradual deterioration of the labyrinth function, which may help to handle the acute unilateral vestibular loss after surgery impacting on the vestibular organ or nerve.

This study aims to systematically review the literature on the effects of ITG pretreatment on the objective vestibular function and subjective outcomes of patients undergoing microsurgery for a unilateral VS.

## Methods

This study was exempt from a review board approval because it involves a systematic review. A literature search was performed in accordance with the Preferred Reporting Items for Systematic Reviews and Meta-Analyses (PRISMA) guidelines. The study protocol was registered in PROSPERO CRD42022309269.

### Search and selection

A search was performed to collect all relevant articles in Pubmed, Embase, Cochrane, Web of Science and Emcare from inception until March 2023. The query consisted of the combination of the following concepts: “unilateral vestibular schwannoma”, “intratympanic gentamicin”, and “objective and subjective vestibular tests”. Two independent authors (CFB and BE) screened titles and abstracts for eligibility. The inclusion criteria comprised the use of ITG, unilateral VS patients, and one or more objective vestibular function tests. If the article also presented subjective vestibular tests, i.e., dizziness-related or quality-of-life questionnaires, these data were also analyzed.

**Fig. 1:**
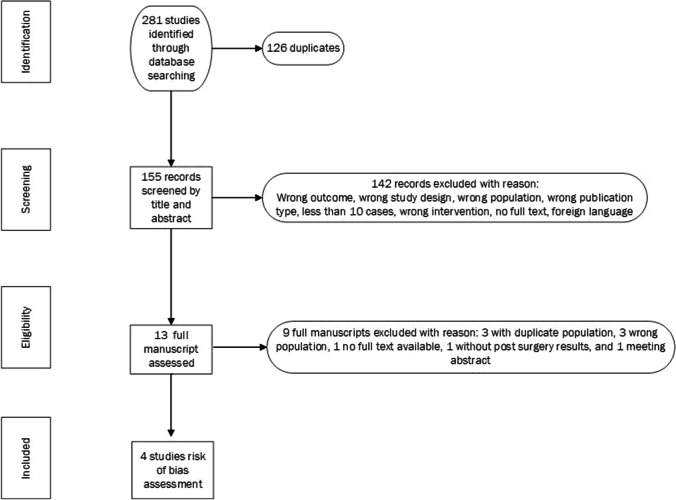
Flowchart for the selection of studies on the effects of intratympanic gentamicin (ITG) in the vestibular function of vestibular schwannoma patients

The exclusion criteria comprised age < 18 years, animal studies, and case reports or series involving less than 10 patients. Just studies in English were included (see Fig. 1).

Two independent reviewers (CFB and BE) screened the full texts of eligible articles. If the full text was not available and/or study characteristics remained unclear after full-text screening, those authors were contacted by email.

### Data collection and analysis

Data on study design, sample size, age, sex, VS treatment, mean tumor size, ITG regimen, balance exercises (vestibular rehabilitation), mean follow-up, objective vestibular tests, dizziness-related, and quality-of-life questionnaires were extracted (when available).

The risk of bias was determined using the Methodological Index for Non-Randomized Studies (MINORS) [7]. This tool contains eight items for assessment of non-comparative studies and four extra questions for comparative studies. The items are scored 0 if not reported, 1 if reported but not adequate, and 2 if reported and adequate. The ideal global score is 16 points (8 items) for the non-comparative studies and 24 points (12 items) for the comparative studies [7]. A low risk of bias was defined as a score between 12 and 16 for the non-comparative studies, and between 20 and 24 for the comparative studies. A high risk of bias was defined as a score ≤ 12 for non-comparative and ≤ 20 for comparative studies [8].

We looked into methodological and statistical heterogeneity. If the data were too heterogeneous for pooling based on methodology and statistics, we performed a descriptive review and summarized the available data. Study selection, data extraction, and quality assessment were performed by two independent reviewers (CFB and BE). If necessary, disagreements were discussed with a third reviewer (EH).

## Results

### Search and selection

A total of 281 articles were identified, of these 126 were duplicates. Titles of 155 unique references were screened, of which 142 records were excluded because of: wrong population, wrong publication type, inclusion of less than 10 cases, wrong intervention, no full text available or non-English language. The remaining 13 studies were full text reviewed for eligibility. We noticed overlapping study populations in 4 articles from Tjernström et al [3, 9–11]. After contacting the authors, the finding was confirmed and the authors referred us to their last study from 2019 [9]. Of the remaining 10 articles, six were excluded (three because of the wrong study population, one without availability of the full text, one that only presented pre-surgery data confirming vestibular dysfunction with the video head impulse test, and one meeting abstract).

Finally, 4 studies were included in the analysis of the risk of bias [9, 12–14] (see Fig. 1).

### Risk of bias

The results on risk of bias are shown in Table 1.

**Table 1:** Risk of bias assessment of the selected studies

Author, year	A clearly stated aim	Inclusion of consecutive patients	Prospective collection of data	Endpoints appropriate to the aim of the study	Unbiased assessment of the study endpoint	Follow-up period appropriate to the aim of the study	Loss to follow-up less than 5%	Prospective calculation of the study size	An adequate control group	Contemporary groups	Baseline equivalence of groups	Adequate statistical analyses	Total Score	Risk of bias (low/ high)
Fellmann et al. [12]	2	2	1	2	0	2	1	0	NA	NA	NA	NA	10/16	High
Balatkova et al. [14]	2	0	0	2	0	2	0	0	2	2	1	2	13/24	High
Hrubá et al. [13]	2	2	2	2	0	2	1	0	2	2	1	2	18/24	High
Tjernström et al. [9]	2	2	1	2	0	2	2	0	2	2	1	2	18/24	High

Grading studies on relevance and validity: 2 = reported and adequate; 1 = reported but inadequate; 0 = not reported. Total ideal score is 16 for non-comparative studies and 24 for comparative studies

By means of the MINORS tool, three studies were scored with a high risk of bias on the inclusion of consecutive patients and reporting the loss of follow-up [9, 12, 13]. In all four studies, there was a low risk of bias on the specification of the study aim, appropriate study endpoints, and appropriate follow-up period for all the studies [9, 12–14].

Overall, all included studies were classified as having a high risk of bias. Based on the great diversity in vestibular outcome assessments, differences in therapeutic regimen and follow-up, it was not justifiable to pool the data from these studies [9, 12–14].

### Data collection and analysis

All four included studies used the ITG as part of a prehabilitation program [9, 12–14]. Study characteristics of the four included studies are shown in Table 2.

**Table 2:** Study characteristics of selected studies

References	Design	Sample size (*n*)	♂ (%); Mean age ± /(range)	Vestibular schwannoma treatment	Mean tumor size—mm	Intratympanic gentamicin regimen	Balance exercises (physical training)	Mean follow-up	Objective vestibular tests	Dizziness-related and QoL questionnaires	Control group
Fellmann et al. [12]	Retrospective study	68	41 (60%); 49.6 (SD 11.5)	Surgery; translabyrinthine (38%), retrosigmoidal (52%), transtemporal (10%) approach	20.2 (SD 9.4)	29 patients under gentamicin No dosage given	Yes	1 year	FGA	DHI	Yes
Balatkova et al. [14]	Retrospective study	32	13 (41%); 47.12 (20–67)	Surgery; retrosigmoidal approach (100%)	Koos classification 2 = 9 patients 3 = 4 patients 4 = 19 patients	3 injections of 0.5–1 ml (40 mg/ml) 2–3 weeks in between injections	Yes	1 year	SVV Optokinetic nystagmus Spontaneous nystagmus Caloric test	GBI GHSI DHI GAD-7 ZUNG	Yes
Hrubá et al. [13]	Prospective study	52	21 (40%); 47.9 (SD 13.2)	Surgery; retrosigmoidal approach (100%)	22.1 (SD 10.6)	3 injections of 0.3–0.6 ml (40 mg/ml) During one day, 2 h between instillation) With a max. of 6 doses in some cases	Yes	14 days	Caloric test Posturography SVV	ABC	Yes
Tjernström et al. [9]	Retrospective study	57	28 (49%); 52.2 (SD 11.4)	Surgery; translabyrinthine (50%), retrosigmoidal (52%) approach	18.3 (SD 7.4)	1–4 injections of 0.5–1.0 ml (30 mg/ml)	Yes	6 months	Posturography SVH	No	Yes

*FGA* Functional gait assessment, *DHI* dizziness handicap inventory, *SVV* subjective visual vertical, *GBI* Glasgow benefit inventory, *GHSI* Glasgow health status inventory, *GAD-7* Generalized anxiety disorder assessment, *ZUNG* Zung self-rating depression scale, *ABC* Activities-specific balance confidence scale

All the included articles were observational and comparative studies [9, 12–14]. All the articles made a distinction between the group of patients who received ITG before the microsurgery, and the group of patients (control group) who did not get ITG as part of the prehabilitation strategy before microsurgery. One study mentioned that the ITG was administered 2 months before surgery [14]. The remaining studies did not provide information on the time interval between ITG and surgery [9, 12, 13]. Regarding the vestibular function in the control groups, Fellmann et al. reported that the patients in the control group had no residual vestibular function on the ipsilesional side. Balatkova et al. did not give information on the vestibular function. Hrubá et al. reported that the control group had vestibular hypofunction on the tumor side, and Tjernström had two groups that did not receive ITG, one group with and one group without vestibular function before surgery [9, 12–14].

Three studies were retrospective [9, 12, 14] and one prospective [13]. All of them involved microsurgery treatment of the VS, either via a translabyrinthine or retrosigmoidal approach. The total number of participants was 209 (male: 49.3%), and the mean age was 49.2 years (SD 12). The mean tumor size was 20.2 mm (SD 9.1) in three of the studies [9, 12, 13]. The remaining study used the Koos classification to grade the tumor size [14]. Patients were included if they had a unilateral VS, received gentamicin injections previous to microsurgery and had at least one objective vestibular test after surgery. In addition to ITG, all study protocols involved vestibular rehabilitation exercises provided by specialized physiotherapists to enhance their balance [9, 12–14].

Tables 3 and 4 outline the summary of objective vestibular outcomes and dizziness-related and quality of life questionnaires outcomes, respectively.

**Table 3:** Summary of objective vestibular function test

References	Vestibular test	Results								Conclusions
		ITG mean (SD)			*P* value	Control mean (SD)			*P* value (ITG vs. control)	
Fellmann et al. [12]	FGA	Pre 27 (23–28)	Post 26 (24–28)		*p = *0.99	26 (25–27)			*p = *0.60	ITG had no effect on postural stability
Balatkova et al*.* [14]	SVV	Time 1 = 0.00 (0.00) Time 2 = 2.50 (4.09) Time 3 = 0.00 (0.00)				Time 1 = 0.00 (0.00) Time 2 = 1.33 (1.72) Time 3 = 0.92 (1.75)			Time 1: *p = *1.000 Time 2: *p = *0.911 Time 3: *p = *0.152	ITG group had abnormal results compared to control group one week after surgery. But the results were similar after the 14 days for both groups
	Optokinetic nystagmus (30°)	Time 1 = L 0.33 (0.19) – R 0.39 (0.24) Time 2 = L 0.44 (0.17) – R 0.35 (0.13) Time 3 = L 0.26 (0.14) – R 0.34 (0.21) Time 4 = L 0.32 (0.18) – R 0.34 (0.16)			–	Time 1 = NR Time 2 = L 0.18 (0.10) – R 0.17 (0.08) Time 3 = L 0.27 (0.15) – R 0.18 (0.21) Time 4 = L 0.34 (0.22) – R 0.30 (0.16)			*p = *0.065	ITG group had higher gain compared to control group. Less sensitive to movement
	Spontaneous nystagmus	Time 1 = 0.0 (0.00) Time 2 = 0.11 (0.33) Time 3 = 0.10 (0.32) Time 4 = 0.0 (0.00)			–	Time 1 = 0.08 (0.28) Time 2 = NR Time 3 = 0.38 (0.51) Time 4 = 0.25 (0.45)			Time 1: *p = *0.358 Time 2: *p = *NR Time 3: *p = *0.132 Time 4: 0.096	After ITG, patients presented with spontaneous nystagmus. Control group did not However, no statistical significant difference
	Caloric test (canal paresis)	75–90% 10–58%			–	0–39%			–	–
Hrubá et al. [13]	Vestibular test	Base	Post-ITG	Post-rehab	*P* value	Base	Post-surgery	Post-rehab	*P* value (ITG vs. control)	Conclusions
	SVV	2.3°	4.6°	3°	–	0.7°	4.7°	3.3°	–	SVV deviation was bigger in ITG group. However, similar results in both groups 2 weeks after rehabilitation
	Posturography CoP (mm^2^)	4592	7656	5662	–	3090	6668	4305	–	No statistical significance between the groups
	Caloric test (CP)	26.7 ± 15.2	32.43	–	–	28.9 ± 23.1	25.25	–	*p = *0.73 (before surgery)	ITG had no effect on caloric test results
Tjernström et al. [9]	Vestibular test	Pre/post (p value)	Group x pre/post (*p* value)		P value	Pre/post x vision (*p* value)	Group x pre/post x vision (*p* value)		Conclusions	
	Posturography Quiet stance	G1 vs. G2: 0.777 G1 vs. G3: 0.523 G2 vs. G3: 0.667	0.883 0.425 0.368		–	0.195 0.952 0.405	0.388 0.606 0.253		Better postural performance in ITG group	
	Vibratory perturbation	G1 vs. G2: 0.479 G1 vs. G3: < 0.001 G2 vs. G3: 0.430	0.010 0.903 0.010		–	0.181 0.470 0.113	0.271 0.654 0.508		Less visual dependency in patients with remaining vestibular function (G2) compare with patients with no vestibular function (G1)	
	SVH	G1 vs. G2: 0.006 (8.4) G1 vs. G3: 0.049 (4.1) G2 vs. G3: < 0.001 [18.5]	0.04 (9.5) 0.027 (5.2) 0.154 (2.1)			NA	NA		Significantly larger visuospatial errors in ITG group (G3) compared to group of patients with remaining vestibular function (G2)	

*ITG* intratympanic gentamicin, *FGA* Functional gait assessment, *SVV* subjective visual vertical, *L* left, *R* right, *CP* canal paresis, *CoP* center of foot pressure, *G1* no vestibular function, *G2* vestibular function, *G3* patients under ITG + vestibular rehabilitation, *SVH* subjective visual horizontal

**Table 4:** Summary of dizziness-related and QoL questionnaire

References	Test	Results								*p* value between groups	Conclusions
		ITG mean (SD)				Control mean (SD)					
Fellmann et al. [12]	DHI total	Pre 2 (0–25)		Post 14 (7–30) *p = *0.14		Pre Entire cohort 10 (0–25)		Post Entire cohort 6 weeks: 23 (5–29) *p = *0.03 1 year: 12 (6–27) *p* > 0.99		*p = *0.07	No statistical difference between groups ITG had no effect on DHI total score
Balatkova et al*.* [14]	GHSI total	Time 1: 64.03 (13.39) Time 2: 60.83 (11.30) Time 3: 56.23 (15.36)				57.18 (15.87) 48.18 (10.7) 46.16 (10.44)				0.264 0.014 0.093	No statistical differences between ITG and control in DHI, GAD-7 and ZUNG
	GBI total	Time 2: 3.92 (5.75) Time 3: 8.02 (11.82)				– − 6.55 (14.84)				– 0.039	
	DHI total	Time 1: 12.20 (13.93) Time 2: 15.40 (13.37) Time 3: 18.60 (21.87)				20.13 (21.86) 31.73 (25.29) 35.87 (25.84)				0.451 0.164 0.191	
	GAD-7	Time 1: 2.56 (3.84) Time 2: 2.78 (3.67) Time 3: 1.22 (1.64)				4.19 (5.00) 4.69 (5.36) 4.88 (4.91)				0.454 0.404 0.020	
	ZUNG	Time 1: 31.33 (6.69) Time 2: 28.11 (11.98) Time 3: 28.67 (13.09)				35.82 (11.79) 36.24 (14.79) 36.82 (14.01)				0.500 0.145 0.117	
Hrubá et al. [13]	ABC	Pre 91	Post 63		Post rehab 81	Pre 92	Post 60		Post rehab 80	–	Significant difference before and after surgery and rehabilitation. ITG had no effect on ABC score No difference between the groups

*DHI* dizziness handicap inventory, *ITG* intratympanic gentamicin, *GBI* Glasgow benefit inventory, *GHSI* Glasgow health status inventory, *GAD-7* Generalized anxiety disorder assessment, *ZUNG* Zung self-rating depression scale, *ABC* Activities-specific balance confidence scale

### Results of objective vestibular tests

#### Spontaneous nystagmus

One study presented results on the observation of spontaneous nystagmus. No significant differences between the ITG pretreatment and control groups were found, 1 year after surgery [14].

#### Optokinetic nystagmus (OKN)

One study reported on OKN with a follow-up of one year after surgery. The gain of the OKN at baseline was higher in the ITG group than in the control group. Patients who underwent gentamicin treatment were more resistant to OKN stimulation (*p = *0.065), which can be interpreted as less sensitive to movement [14].

#### Posturography test

Two studies reported on posturography tests. Hrubá et al*.* showed no significant differences when comparing the ITG group to the control group, after a follow-up of 14 days. Tjernström et al*.* divided the patients into three groups according to vestibular function before microsurgery: one group with no vestibular function at baseline, a second group with normal vestibular function (fast deafferentation), and a third group with normal vestibular function that received ITG before surgery (slow deafferentation). They reported that during quiet stance, patients with vestibular function who did not receive ITG prior surgery performed significantly worse during posturography with eyes closed compared to the patients with no vestibular function. During vibratory perturbation test after surgery, patients in the ITG group and in the “no vestibular function” group had significantly better stability than the group of patients with residual vestibular function before surgery, at 6-month follow-up [9, 13].

#### Postural stability

Postural stability was evaluated using the Functional Gait Assessment (FGA) test. Fellmann et al. found no significant effect on the postural stability between patients with and without ITG pretreatment, 6 weeks and one year after surgery (*p = *0.60) [12].

### Subjective visual vertical (SVV) test

Contrary to what the name suggests, the subjective visual vertical test is regarded as an objective vestibular function test. Two studies reported on SVV test [13, 14]. Both identified no significant differences between the ITG and control group, one study after 14 days, and one after 1 year [13, 14].

### Subjective visual horizontal (SVH) test

Just one study reported on SVH results, using the rod and frame test. They showed significantly larger visuospatial errors post-surgery in the ITG group than in the “no vestibular function” group, which authors state may imply that gentamicin had damaged the vestibular function, after a follow-up of 6 months [9].

### Caloric test

Two studies evaluated the effect by means of the caloric test only at baseline. Balatkova et al*.* reported that already 75–90% of the patients presented canal paresis before ITG and surgery. They did not report the outcome of calorics after ITG nor surgery [14]. Hrubá et al*.* showed a mean canal paresis of 26.7 ± 15.2 in the ITG group before treatment and a mean of 28.9 ± 23.1 in the control group. No results after gentamicin treatment were presented [13].

### Dizziness and quality of life

Three studies reported on different tests to assess dizziness-related quality of life [12–14]. Fellmann et al*.* found no significant difference between the ITG group and patients who did not receive gentamicin using the Dizziness Handicap Index (DHI) after a follow-up of one year after surgery [12]. Balatkova et al*.* reported no significant difference between ITG and control group with regards to the DHI, the Generalized Anxiety Disorder Assessment (GAD-7) and the Self Rating Depression Scale (ZUNG) after VS surgery [14]. Hrubá et al. did not find a significant effect of ITG pretreatment on the activities-specific balance confidence scale (ABC) score after surgery [13].

## Discussion

The deafferentation due to the VS surgery will cause an inherent acute unilateral vestibular lesion. This vestibular loss might be reflected in vertigo, difficulties with balance control or dizziness immediately after surgery. In an attempt to reduce the patient’s postsurgical vestibular symptoms, the use of intratympanic gentamicin pretreatment could help to stepwise diminish the ipsilateral peripheral vestibular function before microsurgery, and with this allowing for static vestibular compensation.

The results of this systematic review on the objective vestibular tests showed either a decrease of the vestibular function or no changes in the group of patients that received ITG before microsurgery compared with the group of patients without gentamicin pretreatment. However, studies that also assessed subjective vestibular outcomes through dizziness and quality-of-life questionnaires did not show less or less severe vestibular symptoms after surgery with ITG prehabilitation.

Tjernström et al*.* and Balatkova et al. showed a positive decrease in the objective vestibular function after gentamicin pretreatment. The patients who received ITG prior to microsurgery performed better in the posturography test, SVH test and the OKN test afterwards [9, 14]. Conversely, Fellmann et al*.,* Hrubá et al*.,* and Balatkova et al*.* did not find significant differences in objective vestibular tests (caloric test, spontaneous nystagmus, SVV, and posturography) after surgery between patients that received ITG pretreatment and those that did not [12–14]. The absence of differences in the caloric test outcomes is explained by the fact that after ITG treatment, all patients underwent surgery with transection of the vestibular nerves, resulting in an inherent vestibular areflexia on the affected side. The similar outcome of the spontaneous nystagmus test may be explained by the timing of the measurement during the follow-up period, which was up to one year [14]. It is well known that spontaneous nystagmus will be present in case of an acute vestibular syndrome but disappears in chronic vestibular dysfunction.

The SVV test did not show significant difference between ITG and control groups, at short term (14 days) or long term (1 year). One possible explanation is because the otolith function was already damaged due to the VS, and the gentamicin did not have a (substantial) additional effect on the vestibular function.

It is interesting that Tjernström et al*.* and Hrubá et al*.* report contradictory results of the postoperative posturography test. One explanation may be the differences in the gentamicin dosage*.* Tjernström et al*.* found a positive result of ITG pretreatment, using a dosage regimen of one–four injections of 0.5–1.0 ml (30 mg/ml) which translates into a total dose of 15–120 mg. Hrubá et al*.* failed to identify significant changes in the posturography results after the use of 3 injections with a maximum of six doses of 0.3–0.6 ml (40 mg/ml) adding up to 36–72 mg of gentamicin in total. Another explanation may be the differences in the posturography test methodology and the way the results were reported. Tjernström et al*.* used six sensors to evaluate the posture disturbances and compared 3 groups of patients according to their vestibular function previous to the treatment. Hrubá et al*.* just used three sensors and compared 2 groups of patients (ITG vs. control group) [9, 13].

It has been hypothesized that repeated exposure to a sensory organization test, such as posturography, could allow for a learning effect, diminishing its reliability. There are some authors that report findings suggestive of a learning curve, while others seem to not find such an effect [17–19]. According to Wrisley et al., a subject has to be exposed to at least 5 sessions within a period of two weeks, in order for a learning effect to develop [20]. In the study of Hrubá et al., the posturography test was performed 3 times (before operation, 7 days and 14 days after surgery), and in the study of Tjernström et al., the test was performed 2 times (before operation and at 6 months after surgery) [9, 13]. Thus, in the studies included in this review, a learning effect seems unlikely due to the limited number of sessions and the time intervals between them. When evaluating subjective vestibular complaints and their psychological components, none of the questionnaires used (DHI, GAD-7, ZUNG, and ABC) showed significant differences when comparing patients after ITG pretreatment with control groups [14].

In the study protocols of all four studies, vestibular rehabilitation exercises were part of the prehabilitation treatment before the vestibular schwannoma surgery [9, 12–14]. Vestibular exercises are used to promote the motor training of the vestibular system and the re-programming of the central nervous system to optimize the vestibular function. This allows a slow deafferentation of the vestibular nerve before surgery, which will bring an inherent vestibular lesion [6]. Based on this premise, one would expect that these four studies would show similar outcomes; however, this was not the case. Balatkova et al*.* and Tjernström et al*.* showed an effect on the objective vestibular function (i.e., vestibular function loss) with the posturography test, SVH test and/or OKN test, in the group of patients after ITG. While Fellmann et al. and Balatkova et al. failed to identify significant changes after ITG compared with control group with the caloric test, spontaneous nystagmus, and/or SVV test. In the case of Hrubá et al*.,* they did not find significant changes in the posturography test [9, 12–14]. These contradicting trends in the outcomes of objective vestibular function tests are indicative of the complexity of the vestibular system and underline the need for a standardized way of measuring the vestibular function in vestibular schwannoma patients. The video head impulse test (vHIT) may be of added value when assessing the status of the vestibular ocular reflex (VOR) and with that the vestibular function of the patient. This test could be included to evaluate the effects of ITG in the VOR, and to assess the differences in pre- and post-treatment vestibular function. In addition, the timing of both objective and subjective vestibular tests may be of critical importance to their outcomes, as the added value of vestibular rehabilitation may not be most pronounced in the period immediately after surgery, when there is acute vestibular loss due to nerve deafferentation, but when a stable vestibular function loss is reached [15].

While Fellmann et al*.,* Hrubá et al*.,* and Cada et al. did not report beneficial outcomes of ITG pretreatment compared to the control group, none of the included studies report detrimental effects of ITG either [12, 13, 16]. Even so, one may well argue that the subjective benefits of a prehabilitation strategy outweigh its effects on objective test outcomes, and since ITG pretreatment may be cumbersome for patients and does involve investment of healthcare resources, it is doubtful whether its use as a prehabilitation technique is justified in vestibular schwannoma patients based on the available evidence at present. The literature, however, is sparse, and our search only retrieved 4 articles, which all suffer from a degree of bias. Future research is needed to evaluate the efficacy of ITG in VS patients, either as a prehabilitation strategy or as standalone therapy for dizziness, preferably using more comparable study designs and a uniform set of outcome measures, both objective and subjective.

## Conclusion

In this systematic review, we find that the posturography test, SVH and the OKN test report a more pronounced deterioration of the peripheral vestibular function after ITG pretreatment in VS patients, when compared to control groups that did not receive ITG, post-surgery. Theoretically, this deterioration is the desired effect and may help alleviate the vestibular symptoms after VS microsurgery. However, subjective vestibular evaluations do not show better resolution of vestibular symptoms after VS surgery of patients pretreated with ITG when compared to controls. However, to fully elucidate the effect of ITG in VS patients, more research is needed, preferably using more uniform objective and subjective outcome measures including the duration of the follow-up (for example, 3–6 months).
